# Assessment of fitness for duty of underperforming physicians: The importance of using appropriate norms

**DOI:** 10.1371/journal.pone.0186902

**Published:** 2017-10-20

**Authors:** Betsy White Williams, Philip Flanders, Elizabeth S. Grace, Elizabeth Korinek, Dillon Welindt, Michael V. Williams

**Affiliations:** 1 Department of Psychiatry School of Medicine, University of Kansas, Clinical Program, Kansas City, Kansas, United States of America; 2 Professional Renewal Center® (PRC®), Lawrence, Kansas, United States of America; 3 Center for Personalized Education for Physicians (CPEP), Denver, Colorado, United States of America; 4 Department of Family Medicine, University of Colorado School of Medicine, Aurora, Colorado, United States of America; 5 Wales Behavioral Assessment (WBA), Lawrence, Kansas, United States of America; University of North Carolina at Chapel Hill, UNITED STATES

## Abstract

**Objective:**

To determine whether population-specific normative data should be employed when screening neurocognitive functioning as part of physician fitness for duty evaluations. If so, to provide such norms based on the evidence currently available.

**Methods:**

A comparison of published data from four sources was analyzed. Data from the two physician samples were then entered into a meta-analysis to obtain full information estimates and generate provisional norms for physicians.

**Results:**

Two-way analysis of variance (Study x Index) revealed a significant main effect and an interaction. Results indicate differences in mean levels of performance and standard deviation for physicians.

**Conclusions:**

Reliance on general population normative data results in under-identification of potential neuropsychological difficulties. Population specific normative data are needed to effectively evaluate practicing physicians.

## Introduction

The assessment of fitness for work is core to occupational health practice [1]. Fitness for work is defined as an evaluation of a worker’s capacity to work without risk to self or others [2]. Such determinations are particularly important for individuals that work in safety sensitive fields. Doctors, like other workers, can experience health conditions, personality issues and/or external stressors that can negatively impact their work performance. Of concern, recent data from the United States and other countries indicate that doctors are under increasing stress and are experiencing high level of occupational burnout, depression and anxiety [3–5]. Additionally physicians are poor at self-care, seeking assistance for themselves or colleagues, treating colleagues and assuming the patient role [1, 6–13].

Physicians have enormous responsibilities to society. To ensure that physicians are able to fulfill those responsibilities, aspiring physicians participate in a highly competitive and selective admissions process and then complete a rigorous course of education, training, and testing. The reasons for this include the complexity and cognitive demands of medical practice, and the level of responsibility associated with being a physician. Measures of the intellectual performance of medical students and physicians on standardized tests of intelligence historically have found that intellectual performance is approximately one to two standard deviations above the mean of the general population [14, 15]. While there has been a great focus on the academic side of medical education and medical practice, historically the importance of self-care, health and wellbeing, an aspect of the core competency area of professionalism, have received less attention as part of the medical education curriculum or through the developmental lifespan of the physician [16].

Unfortunately, a small but significant minority of practicing physicians have performance issues. The nature of underperformance is complex and difficulties with clinical care, behavioral issues, poor attitudes, and poor health can all contribute to performance difficulties. The literature suggests that 6%–12% of practicing physicians can be classified as dyscompetent with perhaps 16% being underperforming [17]. Given 916,264 physicians held active licenses in the United States in 2014[18], between 54,975 and 109,951 practicing physicians could have issues contributing to their being dyscompetent. In terms of services provided, the data suggest that some 55.7–111.4 million office visits per year and 3.7–7.5 million hospital visits are overseen by physicians who upon assessment could be deemed dyscompetent in the United States alone [19].

Individual factors such as the effects of aging, health problems, psychiatric conditions such as depression, anxiety and substance use issues, burnout, fatigue/sleep deprivation, personality characteristics, and external stressors all can negatively impact physician performance and these are common among US workers [11, 17, 20–24]. Proper evaluation and identification of the causes of performance deficiencies are critical for determining appropriate recommendations, including steps for remediation, necessary oversight and additional evaluations [25]. In the United States, the assessment of physicians with possible performance issues may occur as part of an occupational health evaluation, specialized multidisciplinary fitness for duty evaluation and/or a clinical competence assessment. Such evaluations commonly include assessment of physical, cognitive, psychiatric, psychological and social functioning. As these evaluations need to determine whether a physician is permitted to practice, they may lead to legal challenges.

There is a developing literature that indicates that lower than anticipated neuropsychological performance is associated with physician performance difficulties[26–29]. A recent study found that cognitive impairment in physicians is responsible for 57% of adverse medical events, most of which were determined to be preventable [30]. Neuropsychological performance can be associated with a number of psychiatric and health conditions [31]. Other high accountability professionals have used neuropsychological screening measures and measures of intellectual functioning to assist in fitness for duty determinations. Chappelle and colleagues[32] assessed United States Air Force (USAF) pilots on the MicroCog™, a computerized neuropsychological screening test[33] and the Multidimensional Aptitude Battery II, a computerized test of general cognitive functioning [34]. Similar to physicians, the intellectual functioning of Air Force pilots was significantly higher than general population means. The authors concluded, “Population specific normative data are needed to effectively evaluate rated USAF pilots when rendering aeromedical decisions about their psychological disposition [32].”

Similar with pilots, the use of inappropriate normative data in assessments of physicians could result in under-diagnosis of potential problems areas. This can have negative consequences for the identified physician and patients they care for.

Lower than anticipated levels of neuropsychological performance can be associated with mental health issues such as depression, anxiety, or general distress[35–38], a number of medical conditions[31] as well as poor outcomes of remedial training [27, 28, 39]. The literature supports the importance of neurocognitive screening as part of physician fitness for duty and competency evaluations[26, 30, 40, 41] and the importance of using appropriate normative data. Such data on the MicroCog™ have been lacking. Those charged with assessing a physician’s capacity to perform require an understanding of the expected distribution of that performance to accurately determine if potential impairment is present. Particularly in the context of fitness for duty evaluations, which can be highly contentious and litigious, the presence of appropriate norms is critical in making correct determinations about level of performance.

Given the literature on age, education, and neuropsychological test performance, we were interested in determining: 1. Should population-specific normative data be employed when screening neurocognitive function as part of high stakes assessments of physician fitness to practice and/or clinical competency evaluations; and 2. Assuming the analysis was supportive of the need for population specific norms, given the data currently available on the MicroCog™, what would be the most appropriate norms for physicians be determined?

## Methods

In this study, published data on a computerized neuropsychological screening battery, the MicroCog™ were compared. The MicroCog™, which was initially called the Assessment of Cognitive Skills (ACS) was designed to assess cognitive statues changes that might negatively impact job performance of physicians and other professionals [42]. The MicroCog™ has 18 subtests that are grouped to provide indexes of three distinct types. Level 1 Index scores are “… conceptually formed to represent functioning in five respective neurocognitive domains. These are reflected in the following five indexes, Attention/Mental Control, Memory, Reasoning/Calculation, Spatial Processing, and Reaction Time.” As noted in the manual, Level 2 Indexes are aggregations of these ability measures to abstract the processing speed and accuracy components of each of the Level 1 Indexes and form the Information Processing Speed and Information Processing Accuracy Indexes. Finally, the Level 3 Indexes represent global measures of functioning and differ only in the degree of weight given to processing speed: General Cognitive Functioning (weighs speed and accuracy equally) and General Cognitive Proficiency (preferentially weighs accuracy). Thus, the instrument has nine indexes: five in Level 1, two in Level 2, and two in Level 3. It has well documented internal consistency, test-retest reliability, and validity coefficients [43–45].

### Classification of performance

The MicroCog™ manual (page 108) notes that an Average score falls within a range of one standard deviation above the mean to one standard deviation below the mean (i.e., scores of 85–114), while a Low Average score falls between one and two standard deviations below the mean (i.e., scores of 70–84). Below Average is a performance that falls more than two standard deviations below the mean (i.e., scores of 69 and below).

### Data

Data utilized were general norms found in the MicroCog™ Manual as well as a Physician Sample, as reported in Chapter 5 of the MicroCog™ Manual [33]. A second physician sample[29] and data from Air Force pilots[32] were also compared. The original data, as reported, are provided in Table 1, to facilitate comparison.

**Table 1:** Mean scores, standard deviations, and sample size by study.

	Norm Sample (MicroCog™ Norm)			Physicians (Korinek, 2005)			Pilots (Chappelle, 2010)			MicroCog™ Physicians (Pearson, 2004)		
Index	Mean	Std	N	Mean	Std	N	Mean	Std	N	Mean	Std	N
Attention and Mental Control	100.0	15.0	810.0	110.1	9.1	68.0	103.0	12.9	10612.0	105.5	12.2	169.0
Reasoning/ calculation	100.0	15.0	810.0	106.9	12.3	68.0	97.3	13.0	10612.0	112.6	12.4	169.0
Memory	100.0	15.0	810.0	110.4	10.5	68.0	110.6	13.6	10612.0	110.4	11.1	169.0
Spatial processing	100.0	15.0	810.0	108.8	8.3	68.0	106.7	10.3	10612.0	106.0	9.6	169.0
Reaction time	100.0	15.0	810.0	105.9	7.9	68.0	97.7	12.7	10612.0	113.7	11.1	169.0
Information Processing Speed	100.0	15.0	810.0	108.9	11.0	68.0	N/A	N/A	N/A	101.6	13.1	169.0
Information Processing Accuracy	100.0	15.0	810.0	105.9	10.6	68.0	N/A	N/A	N/A	113.1	10.1	169.0
General Cognitive Functioning	100.0	15.0	810.0	N/A	N/A	N/A	N/A	N/A	N/A	109.3	10.8	169.0
General Cognitive Proficiency	100.0	15.0	810.0	109.6	9.1	68.0	N/A	N/A	N/A	108.4	12.7	169.0

#### Sample 1 MicroCog™ normative sample (MNS)

Reported general population norms for the MicroCog™ were drawn from a standardization sample consisting of 810 adults, 90 in each of nine age groups. The age groups were 18–24, 25–34, 35–44, 45–54, 55–64, 65–69, 70–74, 75–79, and 80–89. The sample included equal numbers of men and women in each of the nine age groups. Three racial groups (African American, Hispanic, and White) were sampled in proportion to the U.S. Census. Four geographic regions (Northeast, North Central, South, and West) were represented with proportions from each region based on the U.S. Census. The sample was stratified based on three educations levels: less than high school, high school, and greater than high school. Norms for the highest education level were used in the current analysis.

#### Sample 2 chappelle pilot sample (CPS)

This sample consisted of 10,612 pilot candidates going through medical flight screening [32]. The vast majority were male (91%). Mean age was 22 years (SD = 2.7). The sample was primarily Caucasian (84%) with 4% Hispanic, 2% African American, 6% “other,” and 4% “not reporting.” Participants were either college graduates or enrolled in their 4^th^ year of college. As might be anticipated in an Air Force sample, all participants were physically and psychologically healthy and met standards required for attending pilot training and becoming a rated USAF pilot.

#### Sample 3 korinek physician sample (KPS)

This sample consisted of 68 practicing physicians whose competence was not in question; 60.3% were male, 39.7% were female [29]. Their mean age was 41.9 (SD = 12.5) with a range of 31–76. Almost half were primary care (29; 42.6%), approximately one quarter were surgical specialists (16; 23.5%) and approximately one third were other (23; 33.9%). Physicians were recruited from “a western metropolitan area”, born, educated, and trained in the United States, with English their primary language. Physicians with visual, hearing, or physical impairments that could interfere with performance on the MicroCog™ were excluded.

#### Sample 4 powell physician sample (PPS)

This sample is referred to in the MicroCog™ manual as “A sample of 169 physicians … [33]” No other description is provided.

### Data analyses and comparison

As noted earlier, the MicroCog™ has a total of 9 Indexes that may be reported. The studies employed in this analysis varied as to the number of indexes that each reported. The MicroCog™ Norm Sample (MNS) and Powell Physician Sample (PPS) report all 9 Indexes. The Korinek Physician Sample (KPS) reported 8 of the 9 Indexes, lacking the General Cognitive Proficiency Index. The Chappelle Pilot Sample[32] (CPS) reported only Level 1 Indexes, lacking all of the higher-level Indexes. This is not an impediment to the basis of comparison, as the Level 1 Indexes contain the data from all the subtests. These indexes served as the core of the analysis in this study. The higher indexes are reported in the meta-analysis estimates and in the discussion. Table 1 provides all the indexes as reported by each of the studies analyzed in this report.

The primary analysis was undertaken employing the general linear model to compare means across study samples. This analysis formed the basis for answering the first research question by determining the presence of group-level mean differences. A series of pairwise analyses were undertaken to determine if the various samples differed. To explore these differences more fully, adjusted post-hoc comparisons were undertaken with a Tukey adjustment comparing the KPS data by individual index to that of the PPS data. The analysis employed Prism 6 for Mac OS X, version 6.0f, August 5, 2014 (GraphPad Software, Inc., La Jolla, CA 92037 USA).

The data from the two physician samples (KPS and PPS) were entered into a meta-analysis employing Comprehensive Meta-analysis Version 2.2.064 (BioStat, Englewood, NJ 07631 USA) to obtain full information estimates of the indexes for physicians. These data informed the normative performances for physicians. A random effects model was employed for this analysis as the model’s underlying assumption of effect differences by sample is the better working model for these data[46] based on the significant interaction. The nominal sample size was not adjusted for sample effect size. These estimates were employed in a new two-way analysis as pooled measures of physician performance and compared to the MNS and the CPS (Study x Index) analysis of variance.

IRB review was not required as the data were drawn from already published data.

## Results

**Fig 1:**
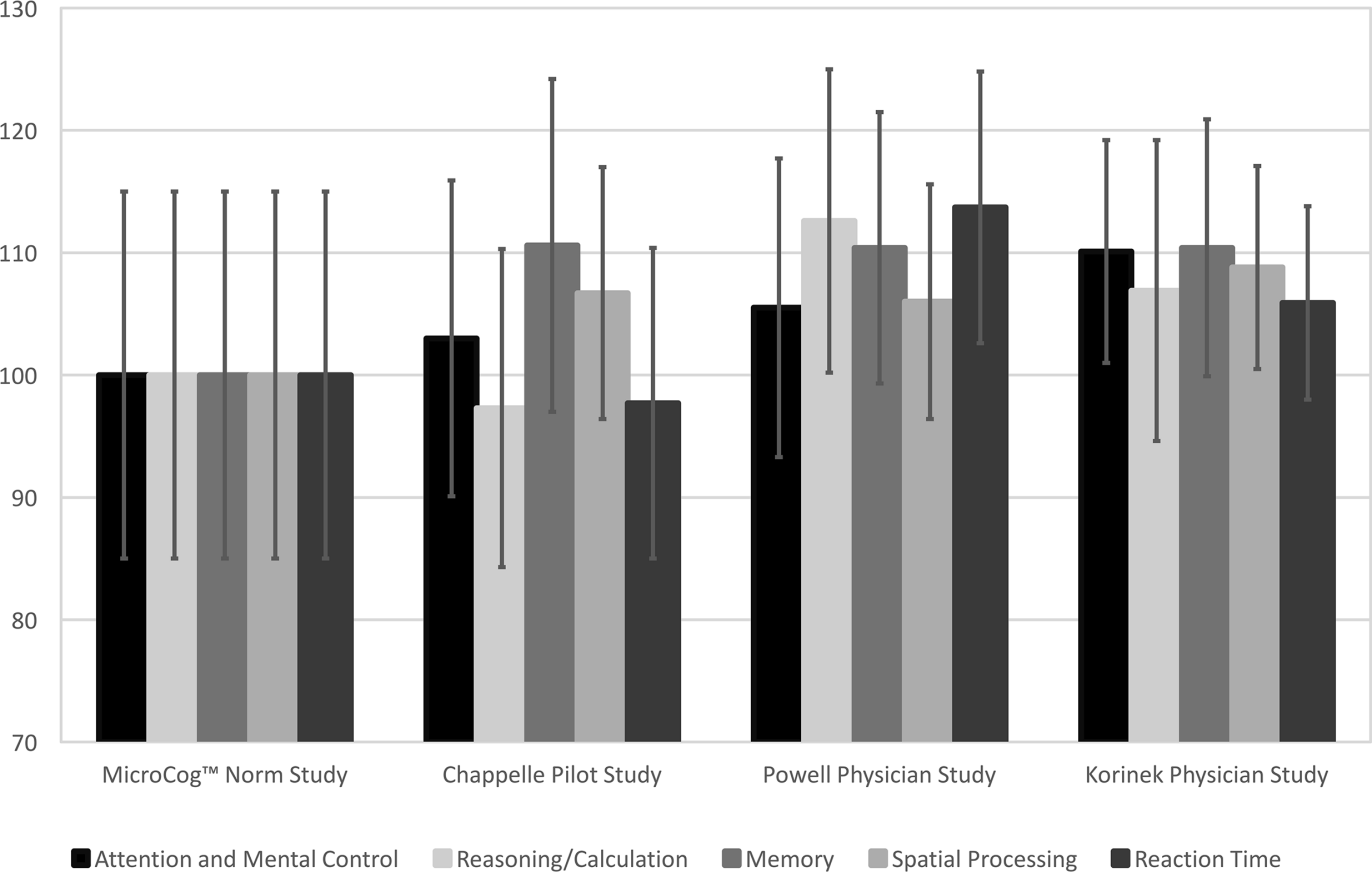
Level 1 indexes by sample. Pattern of mean scores and standard deviations by Study.

Fig 1 provides the Level 1 Indexes in graph form for each of the samples. The marker indicates the mean for each Index and each sample, the standard deviation is indicated by the error bars. When these data are analyzed using a two-way analysis of variance (Sample x Index), the main effect for both Sample and Index as well as the interaction are significant (Study *F*(3, 58275) = 111, *p* < 0.0001, Index *F*(4, 58275) = 27.8, *p* < 0.0001, Interaction *F*(12, 58275) = 9.59, *p* < 0.0001).

When compared to the MNS, each of the three samples was significantly different (KPS *t* = 11.7, *p* < .0001, CPS *t* = 14.8, *p* < .0001, PPS *t* = 19.4, *p* < .0001). When compared to each other, the data from the two physician samples differed significantly from the CPS data (KPS v. CPS, *t* = 7.88, *p* < .0001; PPS v. CPS, *t* = 14.45, *p* < .0001). The data from the two physician samples did not differ significantly from each other (*t* = 1.13, *p* > .259). The effect sizes for these differences are Cohen’s *d* = .77 (KPS) and *d* = .69 (PPS).

To explore these differences more fully, adjusted post-hoc comparisons were undertaken comparing the KPS data by individual index to that of the PPS data. Two of the five indexes proved to be significantly different utilizing a Tukey adjustment. The Attention/Mental Control data in the KPS sample were significantly higher than the PPS data on this index (*t* = 2.78, *p* < .05). On the Reaction Time Index, the KPS data were significantly lower than the PPS data on this index (*t* = 2.72, *p* < .05). The power of the test for the Attention/Mental Control Index was .38, and for the Reaction Time Index was .84. This pattern is the likely basis of the interaction reported above.

The estimates are provided in Table 2 for all the Indexes using the fullest available data for each. Note that the nominal sample size is not adjusted for sample effect size. These estimates were employed in a new two-way analysis as pooled measures of physician performance and compared to the MNS and the CPS (Study x Index) analysis of variance. Both effects were significant (Study *F*(3, 58275) = 111, *p* < 0.0001, Index *F*(4, 58275) = 27.8, *p* < 0.0001). In this case the interaction was not significant.

**Table 2:** Preliminary norms based on meta-analysis of physician performance.

			< -3 sd.	-3 sd.	-2.5 sd.	-2 sd.	-1.5 sd.	-1 sd.	-.5 sd.	.5 sd.	1 sd.	1.5 sd.	2 sd.	2.5 sd.	3 sd.	>3 sd.
Index	Standard Deviation	Mean	*Refer for extended assessment* ….				←←←									
MicroCog			Below Average	Below Average	Below Average	Low Average	Low Average	Average				Above Average				
Heaton			Severe Impairment	Moderate to Severe	Moderate Impairment	Mild to Moderate	Mild Impairment	Below Average	Average		Above average					
Attention/mental control	**11**	**107**	***	73	78	84	90	96	101	113	118	124	130	135	141	***
Reasoning/calculation	**12**	**111**	***	74	80	86	93	99	105	117	124	130	136	142	148	***
Memory	**11**	**111**	***	78	83	89	94	100	105	116	122	127	133	138	144	***
Spatial Processing	**9**	**107**	***	79	84	88	93	98	102	112	116	121	125	130	135	***
Reaction Time	**10**	**112**	***	81	86	91	96	102	107	117	122	127	132	137	143	***
Information processing speed	**13**	**104**	***	66	72	79	85	91	97	110	116	123	129	135	141	***
Information Processing Accuracy	**10**	**111**	***	81	86	91	96	101	106	117	122	127	132	137	142	***
General Cognitive Functioning	**11**	**109**	***	77	82	88	93	99	104	115	120	126	131	136	142	***
General Cognitive Proficiency	**12**	**109**	***	74	80	85	91	97	103	115	121	127	132	138	144	***

## Discussion

The obtained results support that the Norm sample for the MicroCog™ does not effectively represent the pilots or the physician samples. The degree to which these samples vary is uneven across Indexes. Post-hoc comparisons confirm that physicians in these samples perform differently relative to the norm sample reported in the test manual as well as the pilot sample. This finding confirms that physician’s performance is not well represented by the published test norms or norms developed from the pilot sample.

As with Air Force pilots, the use of population specific normative data is critical when making fitness for duty evaluations, competency determinations, treatment, and/or remediation decisions in physicians. The differences between the data drawn from the two physician samples and population MicroCog™ norm sample are even greater than those found between the pilots and the MicroCog™ norm sample. Physicians demonstrated a higher level of performance and less variability around the mean score relative to the published norm group.

While most clinical neuropsychologists adjust their level of expectation based on the common sense notion that a higher level of performance is expected of highly educated individuals on screening tools, such as the Mini Mental Status Exam (MMSE), and the need for the use of appropriate norms for physicians referred for performance issues has also been reported, at this point whether to make such an adjustment, and the level of adjustment, is based solely on each clinician’s professional judgment and experience, making what should be an objective interpretation into a highly subjective one and adjustment to norms is not always made [26, 29, 47, 48]. Also, as the MicroCog^TM^ is used primarily as a screening tool, a clinical neuropsychologist may not always administer and interpret the results.

Comparison of the two physician samples indicated that while they are similar, there appear to be unique differences between them. The reasons for these differences are not clear and raise the possibility of time of measurement effects, demographic variables and/or subtle sub-population differences within the larger physician population. For this reason, when aggregating in the meta-analysis we employed a method with unequal variances. Although the MicroCog^TM^ provides norms for those with some college education, the meta-analysis data presented in Table 2 affirms the need to further adjust for education and provides an evidentiary base for it. In the case of physicians, we suggest that the estimates in Table 2 would provide the most accurate current information on physician performance. In addition to the normally accepted axiom that more data are better than less data, a full information-based estimate of the Indexes is important in that such estimates should more fully approach the population parameters and not be as subject to sampling bias effects as each sample is by itself.

The consequences of misidentification on the MicroCog^TM^ are profound. These consequences could potentially place both the identified practitioner and their patients at risk. With a general population mean of 100 and a standard deviation of 15, compared to a physician population with a mean of approximately 109 and a standard deviation of 11, the likelihood of a Type II error is quite large. A commonly used approach in the context of neuropsychological assessment divides the classification of Average and below into seven different categories with each of the cut off points being one half standard deviation below the next (see Table 2). The score of concern begins at a level of performance that is 1.0 standard deviation below the mean [49]. This corresponds to a score of 85 using the currently published MicroCog™ norms [33]. This cutoff suggests concern for approximately 16% of the general population. However, using the adjusted norms based on this meta-analysis, a score of 85 in the physician population would correspond to a cutoff that is approximately 2.2 standard deviations below the expected mean of a physician cohort. If a normative population score of 85 is used as a cutoff with physicians, over 90% of physicians in whom concerns might be present (the lower 16% of their distribution) would be missed. Put another way, using the meta-analysis norms as the comparison would lead to the identification of approximately nine times as many physicians as potentially suffering from neurocognitive issues. This case study illustrates the importance of appropriate norms (details are provided in a general manner to protect the physician’s identify).

Dr. MD is a 54-year-old Board-certified physician in solo practice. The physician was referred for a fitness for duty evaluation (FFD) secondary to concerns that the physician demonstrated difficulties with interpersonal and communication skills and professional comportment (a “disruptive” physician). The physician completed the evaluation at a private evaluation and treatment center (PETC) specializing in fitness for duty evaluations in high accountability professionals. As part of the multidisciplinary evaluation the physician completed the MicroCog™ as a neurocognitive screen. The results of the Level 1 Indexes, based on age and education corrected norms drawn from the manual are reported below (Table 3). Note that according to the manual scores ranging from 85–114 (16^th^ percentile-82^nd^ percentile) are within the Average range.[33]

**Table 3:** MNS norms vs. meta-analysis norms.

	MNS norms ^33^		Meta-analysis norms	
	Score	%tile	Score	%tile
Attention/Mental Control: Scaled Score	92	30%	92	9%*
Reasoning/Calculation:	97	42%	97	13%*
Memory:	100	50%	100	0.16
Spatial Processing:	87	19%	87	2%*
Reaction Time:	82	12%*	82	0.1%*

*impaired range

Based on the published MNS education corrected norms and using the commonly accepted neuropsychological standard of using one standard deviation below the mean as the threshold for concern (scores of 85 or less), Dr. MD’s results on four of the five Level I index scores are within the Average range. Only one index, Reaction Time falls within the impaired range. Utilizing meta-analysis norms, four of the five scores fall within an impaired range while the fifth falls at the juncture between the Low Average and Mildly Impaired ranges.

Two of the authors (PF and BW) participated in the evaluation and applied the meta-analysis norms in interpreting results. This contributed to the recommendation that the physician undergo comprehensive neuropsychological testing. Those results confirmed neurocognitive deficits and led to recommendations for neurological evaluation prior to return to practice. It was determined that Dr. MD had suffered two recent and previously undetected vascular events and was in need of additional medical treatment prior to return to practice.

A likely course for a physician referred for disruptive behavior not identified as performing poorly on a neurocognitive screening test is referral for educational remediation through continuing medical education.[50] Such an intervention typically would not surface the underlying cause of the behavioral difficulty, in the above case study a vascular event. Of further concern, results of the comprehensive neuropsychological testing completed as part of the fitness for duty evaluation raised the possibility of difficulties in other cognitive areas that led to concerns about issues of clinical competence. Missing the diagnosis could potentially place the doctor’s health and patients at risk.

The rapid expansion of medical knowledge, increases in the complexity of and stressors associated with health care delivery systems, the aging of the physician workforce, increased demand for physician services present challenges to physician health and wellbeing and suggest that physician performance difficulties will continue to be an issue moving forward. This raises the possibility of the increased need for and role of fitness for duty evaluations of physicians. Assessment of fitness for work helps prevent risk to health and safety for the physician, those they work with, and their patients. The importance of competent assessment of doctors’ fitness to work using reliable evidence based tools cannot be cannot be overstated [11, 50]. Given the literature suggesting the potential role neuropsychological factors in physicians with performance issues, accurate identification of potential neuropsychological difficulties in physicians with performance issues is essential to identifying possible health issues, providing appropriate treatment and remediation recommendations, and protecting patient safety. As noted in the recent Assessing Late Career Practitioners: Policies and Procedures for Age-based Screening “…. ideally the screening instrument would be one for which there are published studies conducted with a population comparable to the population of health care practitioners we are working with in this context [51]”

There are a number of limitations in the current study. Comparison of the pilots (CPS) with the MNS group is problematic, due to differences in gender and age distribution. In addition, the two physician samples are small relative to the other study samples. The physician demographic information is unavailable for one physician group (PPS) and limited for the other (KPS), so it is not clear whether the samples are representative of the physician population at large. Both the small sample size and lack of demographic information make it impossible to evaluate for age- or specialty-specific differences among the physicians. As the analysis is based in data that are already age adjusted, age is only a concern if there is an age by profession interaction in neuropsychological performance. Additionally, “Age-adjustment of psychological results, even though standard practice, will underestimate the magnitude of any underlying psychological difficulty in absolute terms, and may be less relevant from a quality assurance viewpoint [28].” There are data from regulatory and academic disciplinary proceedings to suggest that, within the physician community, there are systematic differences in performance on various measures between various subpopulations of physicians [52–54]. Therefore, if there is sampling bias in the two physician samples used, that bias might cause a systematic bias in the classification matrix provided. The direction of any sample-related bias is not known. It is also true that these reference scores do not have access to moments of the distribution beyond the second: this again emphasizes the importance of normative samples beyond those published in the MicroCog™ manual.

The reported physician norms are provided as general guidance, as they are not developed from a true population sample of physicians. However, as the means and variances in the original samples are significantly variant, the pooled, full information, performance estimates will likely more closely reflect the population than either sample alone. Given the high stakes nature of fitness for duty and competency evaluations, meaningful norms with appropriate indicators for further evaluation are critical. The provided data allow some framework and context for making those decisions, as there has previously been little in the literature to support interpretative adjustments on the MicroCog™, should they be challenged. The new norms can provide clarity when dealing with regulatory and legal issues that can often accompany these types of referrals as being able to refer to physician norms can be helpful when needing to justify a disposition on competence to practice, referral for additional medical or neuropsychological testing, a course of remediation, or determination of fitness to practice. In addition, as physicians prefer evidence, physician based norms can be persuasive in encouraging physicians to seek further evaluation and treatment.

It is likely that these proposed norms will result in higher sensitivity in identification of physicians with cognitive difficulties, but may also result in decreased specificity and higher percentage of false positives. Concerns about over-identification of cognitive problems are legitimate. However, the potential impact of lower sensitivity is mitigated by the fact that during physician fitness for duty and competence evaluations, the MicroCog™ is typically used for screening purposes only, and physicians whose performance is below expectations are referred for full neuropsychological testing, thus false positives should be identified through diagnostic testing. While much is at stake for these physicians, it is important to remember that the reason for referral in most cases is to ensure public safety, and therefore it might be most appropriate to err on the side of higher sensitivity and lower specificity of screening. Until such time that more extensive and definitive data are available, the norms provided in this study should not be considered diagnostic, and comprehensive testing should routinely be conducted to further evaluate scores below expectations for physicians.
